# Supplementary material for: Sustained-input switches for transcription factors and microRNAs are central building blocks of eukaryotic gene circuits
Source: Genome Biol. 2013 Aug 23;14(8):R85. doi: 10.1186/gb-2013-14-8-r85 (PMC4054853; doi:10.1186/gb-2013-14-8-r85)
Supplement: Additional file 5 — HTML Browsable Motif Output. Zipped folder containing all WaRSwap and FANMOD motif output, viewable in a web browser. [file gb-2013-14-8-r85-S5.ZIP › HTML_browsable_motif_output/WaRSwap_ath_tair9/sigs_WSWRSLDraw_2-2000.pvals.heatmaps.html/motif_id_46_000101110_tftype_ath_upstream_-3000_0.html]

```
BG_MODEL = Rnew2
MOTIF_ID = 46_000101110
TF_TYPE = ath
UPSTREAM = -3000_0


PVals
FN_0.2	FN_0.4	FN_0.6	FN_0.8
dg_60.genes	0.319	0.001	0.001	0.701
dg_70.genes	0.288	0.002	0.001	0.697
dg_80.genes	0.288	0.002	0	0.692

ZScores
FN_0.2	FN_0.4	FN_0.6	FN_0.8
dg_60.genes	0.53	2.289	3.145	-0.958
dg_70.genes	0.582	2.384	3.263	-0.952
dg_80.genes	0.573	2.274	3.265	-0.973

StDevs
FN_0.2	FN_0.4	FN_0.6	FN_0.8
dg_60.genes	142.431	71.603	30.087	2.536
dg_70.genes	141.446	69.947	29.417	2.444
dg_80.genes	141.811	73.444	29.386	2.367
```
